# 肺癌消化道转移的诊治现状

**DOI:** 10.3779/j.issn.1009-3419.2011.01.14

**Published:** 2011-01-20

**Authors:** 占林 郭, 巴特尔 韩, 宇飞 王

**Affiliations:** 010059 呼和浩特，内蒙古医学院附属医院胸心外科 Department of Cardiotharacic Surgery, The Afliated Hospital of Inner Mongulia Medical College, Huhhot 010059, China

肺癌是最常见的恶性肿瘤之一，恶性程度高。多数患者在就诊时已发生远处转移，常见的转移部位为脑、肝、肾上腺、骨。肺癌消化道转移少见，易出现误诊和漏诊，大多数病例在出现严重的消化道并发症或尸检时才被发现^[1, 2]^。随着肺癌发病率增高、辅助检查日益先进和治疗效果改善，肺癌消化道转移逐渐增多，应引起临床医师的重视，以提高肺癌消化道转移的诊断率。本文就肺癌消化道转移的发生率、诊断、治疗和预后做一综述。

## 1 肺癌消化道转移的发生率

肺癌消化道转移在临床上少见，文献中多以病例报告出现，大宗报道少见。因此，肺癌消化道转移发生率的报道不一，临床发生率约为0.3%-1.77%。Rossi等^[2]^回顾分析了1999年-2006年诊断的原发性肺癌共3 432例，其中18例有消化道转移，平均年龄为68.5岁，男性8例，女性10例，消化道转移发生率为0.5%（18/3, 432）。Yang等^[3]^分析了2003年-2005年诊断的原发性肺癌共339例，其中6例有消化道转移，平均年龄为60.3岁，均为男性，消化道转移发生率为1.77%（6/339）。Lo等^[4]^总结了1994年-2006年行根治性肺切除术的原发性肺癌患者共3 145例，随访发现有10例消化道转移，平均年龄为60.5岁，男性9例，女性1例，消化道转移发生率为0.3%（10/3 145），均出现在术后1个月-25个月。肺癌消化道转移可分为有症状消化道转移和无症状消化道转移，有症状的转移者多在临床上被发现，而无症状者在临床中易被漏诊，多在尸检中被发现。尸检报告的肺癌消化道转移发生率要明显高于临床发生率，且更接近于实际的发生率。Antler等^[1]^复习了1946年-1970年诊断的原发性肺癌尸检资料共423例，其中58例发生消化道转移，平均年龄为61岁，男性54例，女性4例，消化道转移发生率为14%（58/423）。Yoshimoto等^[5]^复习了1972年-2004年诊断的原发性肺癌尸检资料共470例，其中56例发生消化道转移，平均年龄为63.9岁，男性46例，女性10例，消化道转移发生率为11.9%（56/470）。

## 2 肺癌消化道转移的诊断

肺癌消化道转移早期无症状，当消化道转移灶进展出现消化道出血、梗阻或穿孔时才会出现一些相应的症状。食管转移极少见，多数为隆突下淋巴结转移压迫和侵犯食管，或肺原发癌直接侵犯食管。大部分患者无症状，少部分会有进食哽噎感^[1]^。胃转移亦少见，尸检报告的发生率为0.2%-1.7%^[1, 6]^。患者多无症状，有症状者主要表现为上腹部疼痛、呕血、黑便和贫血^[7, 8]^。胃穿孔者极少见，只有2例发生胃穿孔的文献报告^[9, 10]^。在肺癌消化道转移中，以肠道转移最为多见，主要表现为发热、纳差、腹胀、腹痛、排气和排便停止、腹泻、黑便以及进行性的贫血等症状。其中，最常见症状为腹痛^[11]^。在消化道合并症中，最常见的是肠道穿孔^[12]^。2005年，Hillenbrand等^[13]^复习了1961年-2003年文献报道的58例肺癌小肠转移患者也发现消化道穿孔最常见（59%），其次是梗阻（29%）和出血（10%）。消化道穿孔可发生在空肠、回肠、结肠、阑尾等处，空肠和回肠最为常见，尚未发现有十二指肠穿孔的病例；不同的肺癌病理类型导致小肠穿孔的发生率不同，腺癌占23.7%，鳞癌占22.7%，大细胞癌占20.6%，小细胞癌占19.6%^[14]^。有些作者^[9, 15]^推测化疗会诱导消化道转移癌发生坏死穿孔，但消化道转移灶本身的缺血坏死，以及消化道转移灶导致的肠腔梗阻、压力增高可能才是消化道穿孔的主要原因^[16]^。Kim等^[11]^比较了接受过和未接受过化疗的消化道穿孔的患者，发现化疗对消化道穿孔发生率并无影响。此外，Hillenbrand等^[13]^报道的58例小肠转移患者中，有7例是先发现消化道转移而诊断为肺癌，其中5例为消化道穿孔，也说明消化道穿孔与化疗无关。消化道出血多为慢性出血，也有大出血的报道。Steinhart等^[17]^报告了1例十二指肠肺癌转移的消化道大出血患者，胃镜和血管造影均发现肺转移癌侵及十二指肠致十二指肠上动脉大出血，并在随后的尸检中证实。

因症状无特异性，肺癌消化道转移的诊断较为困难。当肺癌患者有上腹部不适、腹痛、呕血、黑便、急性腹膜炎或肠梗阻等消化系统症状时，尤其是出现不能用原发肺部肿瘤或正在进行治疗如放、化疗等解释的腹部症状时，应怀疑有消化道转移的可能。也有研究^[5]^认为，肺癌患者有腹腔脏器转移，特别是肾上腺、肾脏和腹腔淋巴结转移时，容易合并有消化道转移，临床医师应注意是否合并消化道转移。实验室检查及内镜、CT、消化道造影、PET-CT等检查技术有助于诊断^[4, 7]^。Kim等^[11]^对肺癌消化道转移的CT征象进行了研究，28例患者有26例发现了消化道转移的阳性征象，包括局限性肠壁增厚、肠腔内肿物、局部淋巴结肿大、肠套叠和肠穿孔，CT检查可帮助消化道转移的诊断。PET-CT检查在肺癌消化道转移诊断中的应用也有一些文献报道^[18-20]^，对无症状的消化道转移灶检出和肺癌患者的全身转移评估更有意义。

肺癌患者出现了消化道转移的腹部症状，影像学检查有消化道转移的征象，只能高度怀疑有肺癌消化道转移，内镜活检和手术病理检查仍是目前最好的确诊方法。病理诊断除了行常规HE染色外，还应结合已有的原发肺癌病理情况。Rossi等^[2]^发现肺癌消化道转移灶行TTF-l、CK7、CK20、CDX2免疫组化检查有助于明确病理诊断，全组18例患者中，CK20、CDX2均为阴性表达，CK7均为阳性表达，TTF-l有89%的病例为阳性表达，通过这四种免疫组化检查，结合HE染色的结果，可更好判断肿瘤是肺来源或小肠来源。非小细胞肺癌和小细胞肺癌均可出现消化道转移，一部分作者^[1, 6, 11, 21]^认为鳞癌是消化道转移的最常见病理类型，也有一部分作者^[2, 5, 22]^认为分化差的腺癌和大细胞癌是最常见的病理类型。

当肺癌患者出现消化道转移时，多数患者可能已经存在脑、肝、肺、骨、肾上腺等其它脏器的多处转移^[23]^。McNeill等^[6]^报告的46例肺癌小肠转移尸检资料，所有病例至少有2处转移，平均有4.8处转移。Kim等^[11]^报告28例消化道转移的临床病例中，有12例病例有2处以上的转移部位。因此，对肺癌消化道转移患者，若情况允许应在治疗前作全面的检查，以期做出准确全面的诊断。

## 3 肺癌消化道转移的治疗和预后

肺癌消化道转移的处理建议早期发现、早期诊断，可行消化道减状手术，术后全身系统治疗，以改善肺癌患者的生存质量，提高生存率^[12]^。Lo等^[4]^对10例肺癌消化道转移者进行回顾性分析研究，2例胃转移者未手术治疗，8例小肠转移者均行剖腹手术，其中5例行部分小肠切除，小肠对端吻合术，3例仅行剖腹探查活检术，结果显示9例患者生存期未超过3个月，仅有1例小肠转移剖腹探查活检患者生存期为14个月，作者不建议对小肠转移患者行小肠扩大切除和重建。但是，有一些患者因消化道梗阻、穿孔或大出血，则需要行急诊减状手术以挽救生命。在肺癌发生消化道转移患者中，经常会发现消化道有多处转移灶。Antler等^[1]^报告的58例肺癌消化道转移病例中，有3例是肺癌同时转移至胃和小肠，另有3例同时转移至小肠和大肠，2处转移占到全组的10%（6/58）。因此，对剖腹手术的消化道转移患者，一定要仔细探查，以免遗漏转移病灶。截至目前，肺癌消化道转移仍然没有一个适合的治疗方法。

肺癌消化道转移为疾病的终末期，预后差^[12]^。Garwood等^[14]^综合文献报告的98例肺癌小肠转移并穿孔的患者，中位生存期为66天，仅有2例生存时间超过1年。肺癌胃转移似乎比其它部位的消化道转移有更长的生存期^[5]^。也有个别手术治疗后长期生存患者的文献报道。Grenader等^[24]^报告了1例肺鳞癌小肠转移患者经过转移小肠切除重建、原发肺癌射频消融后行肺下叶切除，患者无病生存时间达到2年。Kim等^[25]^报告1例肺癌小肠转移患者手术后无病生存时间已达5年。

## 4 展望

随着肺癌的发病率升高、辅助检查日益先进和治疗效果改善，肺癌消化道转移日渐增多，并不像我们认为的那样少见，因此应引起重视，以提高肺癌消化道转移的诊断率。同时，期望随着肺癌消化道转移治疗经验的积累，能够对肺癌消化道转移进行积极有效的治疗，以改善肺癌患者的生存质量，提高生存率。
